# Factors Associated with Foot Lesions in Diabetic Patients at Saint-Louis Hospital (Senegal): A Case-Control Study Protocol

**DOI:** 10.29337/ijsp.141

**Published:** 2021-04-09

**Authors:** A. Ndong, B. Konta, J. N. Tendeng, D. G. Dia, A. D. Dia, M. L. Diao, A. C. Diallo, S. Diop, B. M. Gouamba, D. A. Dia, M. Diedhiou, M. Dieng, M. L. Fall, P. M. Ma Nyemb, I. Konaté

**Affiliations:** 1 Department of Surgery, Gaston Berger University of Saint-Louis, Senegal; 2 Department of Internal Medicine, Gaston Berger University of Saint-Louis, Senegal; 3 Department of Anaesthesiology, Gaston Berger University of Saint-Louis, Senegal

**Keywords:** diabetes, foot, surgery, amputation, ulcer, gangrene

## Abstract

**Introduction:**

Diabetes prevalence has increased over the past years. In Senegal, this prevalence is 4% in the general population. However, the region of Saint-Louis (in the north of the country) has the highest rate with 10.4%. The main prognosis problem is the occurrence foot lesions that can lead to lower-limbs amputation. Diabetic foot is a real public health issue, due to its economic burden and its serious repercussions on patients, leading to poor quality of life. The objective of this case-control study is to identify factors associated with foot lesions in diabetic patients.

**Methods and analysis:**

It will be a case-control study from January to December 2021. The patients will be recruited from the departments of general surgery, internal medicine, and emergency. An univariate then multivariate analysis (logistic regression) will allow us to select the variables associated with foot lesions in our study population. The parameters included in the logistic regression will be those with a p < 0.20 in the univariate analysis. Finally, a binary logistic regression analysis (with the calculation of Odds Ratios (OR) with confidence intervals (CI)) according to the backward stepwise method will identify the factors independently associated to foot lesions in diabetic patients.

**Ethics and dissemination:**

This research protocol will be submitted to the Ethics Committee of our institution for approval. The knowledge of factors causing diabetic foot will help to communicate with policymakers to raise the awareness in our community. Finally, it will help to prevent lower limb amputations.

**Highlights:**

- Diabetes is the leading cause of non-traumatic lower-limb amputation in the world.
- The region of Saint-Louis (Senegal) has the highest prevalence of diabetes.
- Controlling factors associated with foot lesions in diabetic patients can prevent from amputation.

## 1. Background

Diabetes mellitus is characterized by permanent hyperglycaemia caused by a defect of secretion and/or action of insulin [1]. The prevalence of diabetes has increased over the past years. The estimated number of people with diabetes has increased by 88%, from 246 million in 2006 to 463 million in 2019 [2].

In Senegal, the prevalence of diabetes is 4% in the general population. However, the region of Saint-Louis (in the north of the country) has the highest rate with 10.4% [3]. Type 2 diabetes is the most prominent and represents more than 90% of diabetes and is multifactorial because it results from both a genetic predisposition and reference environmental factors [4,5].

Diabetes is the aetiology of micro and macrovascular complications that lead to foot lesions [6]. Diabetic foot is defined as any infection, ulceration, or destruction of the deep tissues of the foot associated with neuropathy and/or peripheral arterial disease of the lower limbs in diabetic patients [6,7].

Diabetic foot is a real public health issue, due to its economic burden and its serious repercussions on patients, leading to poor quality of life. The main prognosis problem is the occurrence of the lower-limbs amputation [8]. In fact, diabetes is the leading cause of non-traumatic lower-limb amputation in the world (90%) [9,10]. Globally, an amputation is performed every 30 seconds in a diabetic patient [11].

Diabetic foot amputations have increased a lot in the northern region of Senegal, as a total of 778 amputations and disarticulations were performed over a period of 9 years in our hospital [12]. Besides, a previous study also showed that 15.1% of diabetic patient hospitalized had lower limb amputation [13]. It was showed that the risk of amputation is 10 to 30 times greater in the diabetic population than in the general population [14].

The direct economic cost of the diabetic foot is considerable including hospitalization, ambulatory care, amputation and rehabilitation. The indirect cost is also high with social isolation and disabilities [15]. In low-middle income countries, particularly in Sub-Saharan Africa, the cost of care is a major limiting factor to adequate treatment [16]. Studies showed that in most cases, diabetic patients are often diagnosed at stage of complications [17]. Even worse, it was estimated that 75% of diabetic patients are not diagnosed in Africa [18,19]. This late diagnosis is an important prognostic factors that could explain the high rate of diabetic foot and amputations in resource-limited settings.

## 2. Research hypothesis

In the literature, several risk factors to foot lesions in diabetic patients have been described, including neuropathy, peripheral arterial disease, and susceptibility to infections [8,12].

Neuropathy is the main factor due a loss of sensitivity, favouring areas of friction, hyper pressure and static disorders [20]. This finally leads to diabetic nervous osteoarthropathy of Charcot foot. The duration of diabetes and non-adherence to the treatment can intensify the neuropathy [21,22]. Peripheral arterial disease is responsible for ischemia than can lead to ulcers [21]. The high prevalence of arteriopathy in diabetic patient can be explained by the frequency of cardiovascular risk factors (advanced age, alcohol, smoking, arterial hypertension, dyslipidaemia, obesity, sedentary lifestyle) [23,24]. The unfavourable socio-economic level is a factor converging to macrovascular complications of diabetes [4,13].

Determining the risk factors of diabetic foot could help to prevent amputation and its high economic and social burden. Hence, our research question is: “What are the factors associated to foot lesions in patients with type 2 diabetes”?

## 3. Methods and analysis

### 3.1. Objective of the study

The objective of this case-control study is to identify factors associated with foot lesions in diabetic patients.

### 3.2. Study setting

The study will be realised at the Saint-Louis hospital (Senegal). This hospital is the reference center for the northern zone of the country, but also for the southern zone of Mauritania, a border country. Previous studies have found a high prevalence of diabetes in the Saint-Louis region close to 10.4% [25]. A study by our team also showed a rate of amputation of 15.3% [13].

### 3.3. Type and period of study

It will be a single center case-control study from January to December 2021. The patients will be recruited from the departments of General surgery, internal medicine, and emergency.

### 3.4. Study design

This is a case-control study in a hospital setting.

The cases will be defined by adult patients (over 15 years of age) followed for type 2 diabetes with foot lesions regardless of the stage. The University of Texas classification will be used. These patients will be recruited from the general surgery and emergency departments.

The controls will be defined by adult patients followed for type 2 diabetes without any lesion of the foot. These patients will be recruited from the Internal Medicine department.

Patients will be recruited randomly received for consultation or during their hospitalization.

### 3.5. Sampling method

The sample size will be calculated from the BiostaTGV (*https://biostatgv.sentiweb.fr/?module=etudes/sujets#/*). Considering a risk of 5% with a power of 80%, we will take a theoretical exposure in the controls of 30% regarding the high prevalence of diabetes and its complications in the Saint-Louis region. A minimum detectable Odds-Ratio will be set at 0.2. Thus, the sample size will 62 controls versus 31 cases or two controls for one case.

### 3.6. Data analysis

The qualitative variables will be described in number with their proportion and the quantitative variables in the form of mean with their standard deviation.

Univariate analysis will be done to determine the factors associated with the presence of foot lesions in diabetic patients. For the qualitative variables, the statistical tests used will be Pearson or Fischer Chi-square. For quantitative variables, Student’s or Mann Whitney’s t-test will be used. The difference will be considered significant when p < 0.05. The odds ratio (OR) surrounded by its confidence interval (CI) will measure the strength of the association.

The univariate analysis will allow us to select the variables associated with foot lesions in our study population. The parameters included in the logistic regression of the multivariate analysis will be those with a p < 0.20 in the univariate analysis. Finally, we will proceed by binary logistic regression analysis (with the calculation of OR with CI) according to a backward stepwise method to identify the factors independently associated to foot lesions in diabetic patients. The results will be expressed with a 95% CI.

### 3.7. Data collection and entry

Data collection will be prospective on a survey form, entered into SPSS 26 software where statistical analyses will be done. Graphs and tables will be made in Excel.

### 3.8. Studied parameters

The studied parameters will be:

- Epidemiological data: age, gender, profession, address, level of education;
- Clinical data: consultation delay, duration of diabetes, existence of diabetes follow-up, family history of diabetes, dyslipidemia, alcohol and tobacco use, depigmentation, contraception, traditional treatment, footcare, use of suitable shoes, blood pressure, systolic pressure index, temperature, heart rate, respiratory rate, weight, height, body mass index, waist circumference;
- Paraclinical data: fasting blood glucose, HbA1c, hemoglobin (anemia), hematocrit (hemoconcentration), white blood cell (leukocytosis), bacteriology, creatinine level, peripheral arterial doppler ultrasonography, radiography, electrocardiogram;
- Therapeutic data for diabetes: type of treatment (diet, oral antidiabetic drugs, insulin).

Peripheral neuropathy will be assessed by clinical signs (paraesthesia, unconscious shoe loss, and the Semmes-Weinstein 10 mg monofilament test).

Arteriopathy will be assessed by the Leriche and Fontaine classification (palpation of peripheral pulses, systolic pressure index and Doppler echo if available) [23].

For cases, the University of Texas classification will be noted [26]. It will allow to be classify the lesions according to the existence of an infection (presence of pus and/or bacteriology), the existence of ischemia (peripheral pulse, systolic pressure index, Doppler ultrasound of the lower limbs), and the depth of lesions (clinical and/or standard radiography).

### 3.9. Ethics and dissemination

For ethical reasons, we will respect the guarantee of anonymity and confidentiality of information collected in patient. Patients informed consent will be obtained before inclusion. This research protocol will be submitted to the Ethics Committee of our institution for approval.

The results of this study will be presented to national and internationals conferences and published in peer-reviewed journals. Besides, the knowledge of factors causing diabetic foot will help to communicate with policymakers to raise the awareness in our community. Finally, it will help to prevent lower limb amputations.

## Registration of research studies

We have registered our study with unique identifying number: researchregistry6402.
